# Gastrodin attenuates angiotensin II-induced vascular contraction and MLCK/p-MLC_2_ pathway activation

**DOI:** 10.1080/13880209.2023.2207591

**Published:** 2023-05-21

**Authors:** Zhi Guo, Xuan Yang, Meizhu Wu, Aling Shen, Jiapeng Li, Xiuli Zhang, Ying Cheng, Qiurong Xie, Jun Peng

**Affiliations:** a Academy of Integrative Medicine, Fujian University of Traditional Chinese Medicine, Fuzhou, China; b Fujian Key Laboratory of Integrative Medicine on Geriatrics, Fujian University of Traditional Chinese Medicine, Fuzhou, China; c Fujian Collaborative Innovation Center for Integrative Medicine in Prevention and Treatment of Major Chronic Cardiovascular Diseases, Fuzhou, China; d Department of Physical Education, Fujian University of Traditional Chinese Medicine, Fuzhou, China

**Keywords:** Hypertension, vascular smooth muscle cells

## Abstract

**Context:**

Gastrodin has been used as antihypertension therapy in China; however, the mechanisms underlying the effects of gastrodin have yet to be fully elucidated.

**Objective:**

To explore the therapeutic efficiency of gastrodin as an antihypertensive and determine the mechanisms underlying this effect.

**Materials and methods:**

C57BL/6 mice were continuously administered angiotensin II (Ang II) (500 ng/kg/min) to induce hypertension. Mice were randomly divided into control, Ang II and Ang II + gastrodin groups. Mice received intragastric administration of gastrodin (5 mg/kg) or double distilled water once a day for 4 weeks. Blood pressure, pulse wave velocity (PWV), thickness of the abdominal aorta, pathological morphology and differential expression transcripts (DETs) were assessed. Abdominal aorta rings and primary isolated vascular smooth muscle cells were subjected to Ang II stimulation to induce hypertension as *ex vivo* and *in vitro* models, respectively. Vascular ring tension, release of Ca^2+^ and levels of proteins involved in the myosin light chain kinase (MLCK)/phospho-myosin light chain 2 (p-MLC_2_) pathway were determined.

**Results:**

Gastrodin treatment attenuated increases in blood pressure, PWV and thickness of the abdominal aorta. Treatment with gastrodin resulted in 2785 DETs and the enrichment of vascular contraction and calcium signalling pathways. Gastrodin treatment attenuated Ang II-induced vasoconstriction, produced a norepinephrine-precontracted vasodilation effect (attenuated by verapamil), and reduced intracellular Ca^2+^ release. Furthermore, gastrodin suppressed activation of the MLCK/p-MLC_2_ pathway *in vivo* and *in vitro*.

**Conclusions:**

Gastrodin treatment lowers blood pressure, suppresses Ang II-induced vascular contraction and MLCK/p-MLC_2_ pathway activation, thereby demonstrating the mechanisms underlying the therapeutic efficacy of gastrodin as an antihypertensive.

## Introduction

Hypertension is a progressive cardiovascular syndrome affecting over 1.4 billion individuals globally (Rossier et al. 2017; Oliveros et al. 2020). Continuous increases in blood pressure lead to changes in vascular function and structure over time (Chobanian 2017). Pathological changes in blood vessels are closely associated with the development of hypertension, hypertension-mediated organ damage and cardiovascular events (Rizzoni et al. 2023). Accordingly, there is a clinical need for effective antihypertensive medications that provide cardiovascular protection for the management of hypertension.

Excessive activation of the renin–angiotensin–aldosterone system (RAAS) contributes to the initiation and progression of hypertension (Romero et al. 2015; Te Riet et al. 2015; Rossier et al. 2017; Schutten et al. 2017). Angiotensin II (Ang II) is the primary active component of the RAAS. Ang II binds to angiotensin type 1 receptor (AT1R) and triggers downstream signalling involving highly specific fluctuations in Ca^2+^ signalling, thereby resulting in contraction of vascular smooth muscle cells (VSMCs) and elevation of blood pressure (Te Riet et al. 2015; Bennett et al. 2016). As an important secondary messenger, intracellular Ca^2+^ is essential for vascular smooth muscle contraction (Harraz and Jensen 2021). In VSMCs, increased intracellular Ca^2+^ levels induce contraction by binding to calmodulin (CaM) and then activating myosin light chain kinase (MLCK) and phosphorylating the Ser18 and Thr19 residues of myosin light chain 2 (MLC_2_) (Kim et al. 2008; Touyz et al. 2018). Phosphorylated myosin binds to actin fibres, leading to fibre contraction and changes in vessel tension and vascular diameter (Bravo-Sagua et al. 2020). Accordingly, the inhibition of the MLCK/phospho-myosin light chain 2 (p-MLC_2_) pathway and resulting vascular contraction represents a potential strategy for treating hypertension and vascular dysfunction.

Gastrodin (PubChem CID: 115067) is a major bioactive component extracted from *Gastrodia elata* Blume (Orchidaceae) (Liu et al. 2020). In recent years, gastrodin injections have been used for the treatment of hypertension in China (Liu et al. 2018; Qiao et al. 2021; Lai et al. 2022; Zhou et al. 2022). Gastrodin has been shown to reduce blood pressure in both hypertensive patients and spontaneously hypertensive rats (SHRs) (Zhang et al. 2008; Liu et al. 2015; Qian et al. 2020). Moreover, gastrodin injections can reduce plasma endothelin levels and increase plasma nitric oxide levels in hypertensive patients (Zhang et al. 2008; Qian et al. 2020). However, the regulatory mechanisms underlying the effects of gastrodin have yet to be fully elucidated. The present study therefore aimed to investigate the role of gastrodin in regulating hypertension-associated vascular dysfunction in an Ang II-induced hypertensive mouse model and elucidate the mechanisms underlying these effects using multiple technologies, including RNA sequencing.

## Materials and methods

### Reagents

Gastrodin was provided by Shanghai Yuanye Bio Technology Co., Ltd. (purity ≥98%, Shanghai, China). Antibodies against CaM (cat no. ab2860), AT1R (cat no. ab124505), rabbit IgG (Alexa Fluor^®^ 647; cat. no. ab150079) and Ang II were purchased from Abcam (Cambridge, MA). Dulbecco’s modified eagle medium (DMEM), foetal bovine serum (FBS), trypsin-ethylenediaminetetraacetic acid (EDTA), Fluo-4/AM and bicinchoninic acid (BCA) protein assay reagent kits were obtained from Thermo Fisher Scientific (Waltham, MA). Antibodies against MLCK (cat. no. 48846), p-MLC_2_ (cat. no. 11114) and α-SMA (cat. no. 40482) were purchased from Signalway Antibody LLC (Greenbelt, MD). Cell Counting Kit-8 (cat. no. ktc011001) and antibody against GAPDH (cat. no. Abp57259) were obtained from Abbkine Scientific (Wuhan, China). Antibody against MLC_2_ (cat. no. 3672s) and rabbit secondary antibody (cat. no. 7074) were purchased from Cell Signaling Technology (Danvers, MA). Antigen repair solution (cat. no. MVS-0066), ultra-sensitive™ SP (mouse/rabbit) immunohistochemistry (IHC) kits (cat. no. KIT-9720) and 3,3′-diaminobenzidine (DAB) substrate kits (cat. no. DAB-0031) were purchased from Maixin Biotechnology (Fuzhou, China).

### Animals and experimental protocols

C57BL/6 mice (8-week-old; male) were purchased from SLAC Laboratory Animal Technology Co., Ltd. (certificate ID: SYXK 2020-0002, Shanghai, China). All mice were maintained in a specific pathogen-free laboratory under appropriate humidity (55% ± 5%) and temperature (24 °C ± 2 °C) with a light/dark cycle and fed with a standard chow diet and water. After acclimation for 1 week, mice were randomly divided into one of the following three groups (*n* = 6): control, Ang II or Ang II + gastrodin. Mice in the Ang II and Ang II + gastrodin groups received continuous infusions of Ang II (500 ng/kg/min) via a micro-osmotic pump for a total of 4 weeks. The control group received a continuous infusion with the same amount of saline using the same method. Mice in the Ang II + gastrodin group were intragastrically administered gastrodin dissolved in double distilled water (ddH_2_O) once a day at a dose of 5 mg/kg/d. The dose of gastrodin was converted from that of SHR (Wen et al. 2023) by adjusting the body surface area, according to the Methodology of Pharmacological Experiments. Mice in both the control and Ang II groups were intragastrically administered an equal volume of ddH_2_O. All experiments were conducted with the approval of the Institutional Animal Care and Use Committee of Fujian University of Traditional Chinese Medicine (no. FJTCM IACUC 2021133).

### Blood pressure measurements

Blood pressure was measured prior to the experiment and once a week for a total of 4 weeks by a tail-cuff methodology using the CODA™ noninvasive blood pressure system (Kent Scientific, Torrington, CT). Mice were gently restrained in a chamber and transferred to standard equipment with a heating pad for 10 min. The cuffs were then wrapped around the tail and inflated and deflated to measure the blood pressure. Five pre-cycles were performed. The average blood pressure from 15 readings was recorded as the individual blood pressure for each mouse.

### Ultrasound measurements

Pulse wave velocity (PWV) and thickness of the abdominal aorta were measured using a Vevo 2100 high-resolution ultrasound instrument (FUJIFILM VisualSonics, Toronto, Canada) as previously described (Wu et al. 2021). In brief, the mouse was anesthetized (with isoflurane, 2% induction and 1.5% maintenance) and placed on a heating platform in the supine position. When the heart rate was approximately 450 beats per minute, a 30-MHz probe was longitudinally positioned below the sternum and xiphoid process to acquire images of the abdominal aorta in both B and M modes, which were further processed using Vevo^®^ LAB software to measure PWV and thickness of the abdominal aorta.

### RNA sequencing

RNA sequencing was performed by Capital Bio Technology (Beijing, China) as described previously (Long et al. 2021). According to the manufacturer’s instructions, NEBNext Ultra RNA Library Prep Kits for Illumina (New England Biolabs, Ipswich, MA) were used to construct sequencing libraries. DNA was quantified using KAPA library quantitative kits (KAPA Biosystem, Wilmington, MA). The resulting transcriptome sequencing libraries were conducted with Illumina sequencing platform (Illumina, San Diego, CA). Sequencing quality was assessed using FastQC software to ensure the accuracy and reliability of data. All groups of mice were analysed for differential expression using the DESeq method. Differential expression transcripts (DETs) were used for subsequent Kyoto Encyclopedia of Genes and Genomes (KEGG) pathway analysis.

### Haematoxylin and eosin staining

Abdominal aortic tissues were fixed in 4% paraformaldehyde (for 48 h at room temperature) and embedded in paraffin. Specimens were sectioned into 4 µm slices and deparaffinized in xylene. Followed by washing with phosphate-buffered saline (PBS), tissue slices stained with haematoxylin and eosin (H&E) using a H&E staining kit as previously described (Wu et al. 2021). For each tissue sample, three independent fields were randomly selected and examined at ×400 magnification under an intelligent automated optical microscope (Leica, Wetzlar, Germany).

### Immunohistochemical staining

Tissue slices were washed with PBS and then boiled in 10 mM sodium citrate buffer at 95 °C for 10 min for antigen retrieval. Ultra-Sensitive™ SP (Mouse/Rabbit) IHC kits were used to reduce endogenous peroxidase activity and decrease nonspecific staining. Slices were then incubated with primary antibodies for AT1R (1:200), CaM (1:100), MLCK (1:500), p-MLC_2_ (1:100) or MLC_2_ (1:200) at 4 °C overnight. After washing with PBS three times, tissues were incubated with a biotinylated secondary rabbit/mouse antibody and horseradish peroxidase-labelled streptavidin. Protein expression was visualized using DAB in three randomly selected fields of view for each sample. Staining positivity was analysed using ImageJ software (National Institutes of Health, Bethesda, MD).

### Vascular reactivity experiments

To prepare vessel rings, C57BL/6 mice were anesthetized with isoflurane and abdominal aortas were stripped and cut into rings of 2–3 mm length in cold physiological salt solution (PSS; 130 mM NaCl, 4.7 mM KCl, 1.18 mM KH_2_PO_4_, 1.17 mM MgSO_4_·7H_2_O, 14.9 mM NaHCO_3_, 5.5 mM glucose, 0.026 mM EDTA and 1.6 mM CaCl_2_) saturated with a gas mixture (95% O_2_ and 5% CO_2_). An Automated Multi Myograph System (Danish Myo Technology, Aarhus, Denmark) was used to detect changes in vascular tension. Vessel rings were placed in an organ chamber filled with 5 mL PSS saturated with a gas mixture (95% O_2_ and 5% CO_2_) at 37 °C for 45 min for stabilization. During the stabilization period, PSS was replaced every 15 min (Xiang et al. 2020).

After the rings were stabilized, cumulatively increasing concentrations of gastrodin (0.25, 0.5, 0.75, 1.0, 1.25 or 1.5 mg/mL) were added to the organ chamber for 30 min. In the control group, vehicle was added to the organ chamber instead. The change in tension of each vascular ring was recorded to obtain a cumulative concentration–response curve of gastrodin.

We next determined the effect of gastrodin on the vasodilation of abdominal aortic ring precontracted with norepinephrine (NE). After equilibration, NE (1 μM) was added to induce steady contraction of the abdominal aortic ring. Cumulatively increasing concentrations of gastrodin (0.25, 0.5, 0.75, 1.0, 1.25 or 1.5 mg/mL) were added into the organ chamber (30 min for each concentration). The same volume of vehicle was added to samples in the control group. A concentration–response curves of gastrodin (0.25, 0.5, 0.75, 1.0, 1.25 or 1.5 mg/mL) induced-vasodilation was then plotted.

The effect of gastrodin on the contraction of abdominal aortic rings induced by Ang II was detected by pre-incubating abdominal aortic rings with gastrodin (1.25 mg/mL) for 30 min, followed by Ang II (1 μM) stimulation. The obtained results were recorded as percentages of contraction compared to the absence of gastrodin (control). The change in tension of each vascular ring was also recorded.

To determine the role of Ca^2+^ channels in gastrodin-induced relaxation, abdominal aortic rings were pre-incubated with verapamil (10 μM), an l-type calcium channel blocker, for 30 min. Tissues were then stimulated with NE (1 μM). Gastrodin (0.25, 0.5, 0.75, 1.0, 1.25 or 1.5 mg/mL) was then cumulatively added to chamber. Tissues in the control group were not incubated with verapamil. The change in tension of each vascular ring was also recorded.

### Vascular smooth muscle cell isolation and characterization

Adult male Wistar rats at approximately 6–8 weeks of age and weighing 200–250 g were obtained from Beijing Huafukang Bioscience Co., Ltd. (certificate ID: SCXK 2019-0008, Beijing, China). Primary isolated VSMCs were obtained as previously described (Wu et al. 2021). Rats were anesthetized by isoflurane and abdominal aortas were collected and placed in cool CaCl_2_-HEPES-buffered salt solution (HBSS). Excess fat tissue was removed and aortas were longitudinally cut. Endothelial cells were then gently removed by scraping. The remaining membrane was digested in 0.5 mL of Ca^2+^ free HBSS containing collagenase I (3.2 mg), papain (0.3 mg) and bovine serum albumin (BSA; 2 mg) at 37 °C for 30 min. The acquired VSMCs were cultured in DMEM supplemented with 100 U/mL penicillin, 100 μg/mL streptomycin (Hyclone, Logan, UT) and 10% FBS at 37 °C under 5% CO_2_. Primary isolated VSMCs were characterized by ubiquitous expression of α-SMA (a marker of VSMCs).

### Cell Counting Kit-8 assays

Primary isolated VSMCs were seeded into 96-well plates at a density of 2 × 10^4^ cells/mL and cultured overnight. After starvation for 6–8 h, primary isolated VSMCs were treated with gastrodin at 12.5, 25, 50, 100 or 200 μM or Ang II at 0.01, 0.1, 1 or 10 μM for 24 h. At the end of treatment, 10 μL CCK-8 solution was added to each well and cells were incubated in a cell culture incubator at 37 °C with 5% CO_2_ for 2 h in the dark. The optical density of each well at 450 nm was measured using a microplate reader (Thermo Fisher Scientific, Waltham, MA).

### Measurement of intracellular Ca^2+^ concentrations

Primary isolated VSMCs (4 × 10^4^ cells/well) were pretreated with or without gastrodin (100 μM) for 24 h after deprivation of serum for 6–8 h. Cells were incubated with Fluo-4/AM (5 mM), and changes in intracellular Ca^2+^ concentrations were determined using a confocal microscope (Ultra VIEW^®^ Vox, PerkinElmer, Waltham, MA) according to the manufacturer’s protocol. Samples were scanned in real time for approximately 900 s in Ca^2+^-free HBSS. Ang II (1 μM) was added at 300 s. Fluorescence intensities were normalized to basal levels.

### Western blotting

Cells were lysed with Western and IP cell lysis buffer (Beyotime Biotechnology, Shanghai, China; cat. no. P0013J) containing phenylmethylsulfonyl fluoride (PMSF) (Beijing Solarbio Science & Technology Co., Ltd., Beijing, China; cat. no. P0100), protease inhibitor cocktail (MedChemExpress, Shanghai, China; cat. no. HY-K0022) and PhosStop (Roche, Shanghai, China; cat. no. 4906945001). Samples were then centrifuged at 12,000 × *g* at 4 °C for 20 min. Protein concentrations were determined using BCA kit. Denatured proteins were resolved using 10% sodium dodecyl sulphate polyacrylamide gel and transferred to polyvinylidene difluoride membranes. After blocking with 5% skim milk for 1 h at room temperature, membranes were incubated with diluted primary antibodies against AT1R (1:1000), CaM (1:1000), MLCK (1:5000), MLC_2_ (1:1000), p-MLC_2_ (1:500) or GAPDH (1:5000) at 4 °C overnight. After incubation with secondary rabbit/mouse antibody (1:5000) at room temperature for 1 h, protein expression levels were detected using BeyoECL Plus (Beyotime Biotechnology, Shanghai, China; cat. no. P0018M) and analysed using ImageJ software (National Institutes of Health, Bethesda, MD).

### Statistical analyses

Statistical analyses were performed using SPSS 26.0 software (SPSS/PC+; Chicago, IL). Data were presented as the mean ± standard deviation (SD). Normality of data was determined using the Shapiro–Wilk test. The independent Student’s *t*-test was performed to compare differences between two groups. One-way analysis of variance was used to compare differences between three or more groups when data were normally distributed. Bonferroni’s correction was used when the variance was Chi-square, and the Games–Howell test was used when the variance was not Chi-square. The nonparametric Kruskal–Wallis test was used to compare differences between groups when data were not normally distributed using pairwise comparisons. *p* Values less than 0.05 were considered statistically significant.

## Results

### Gastrodin decreases blood pressure in hypertensive mice

**Figure 1.**
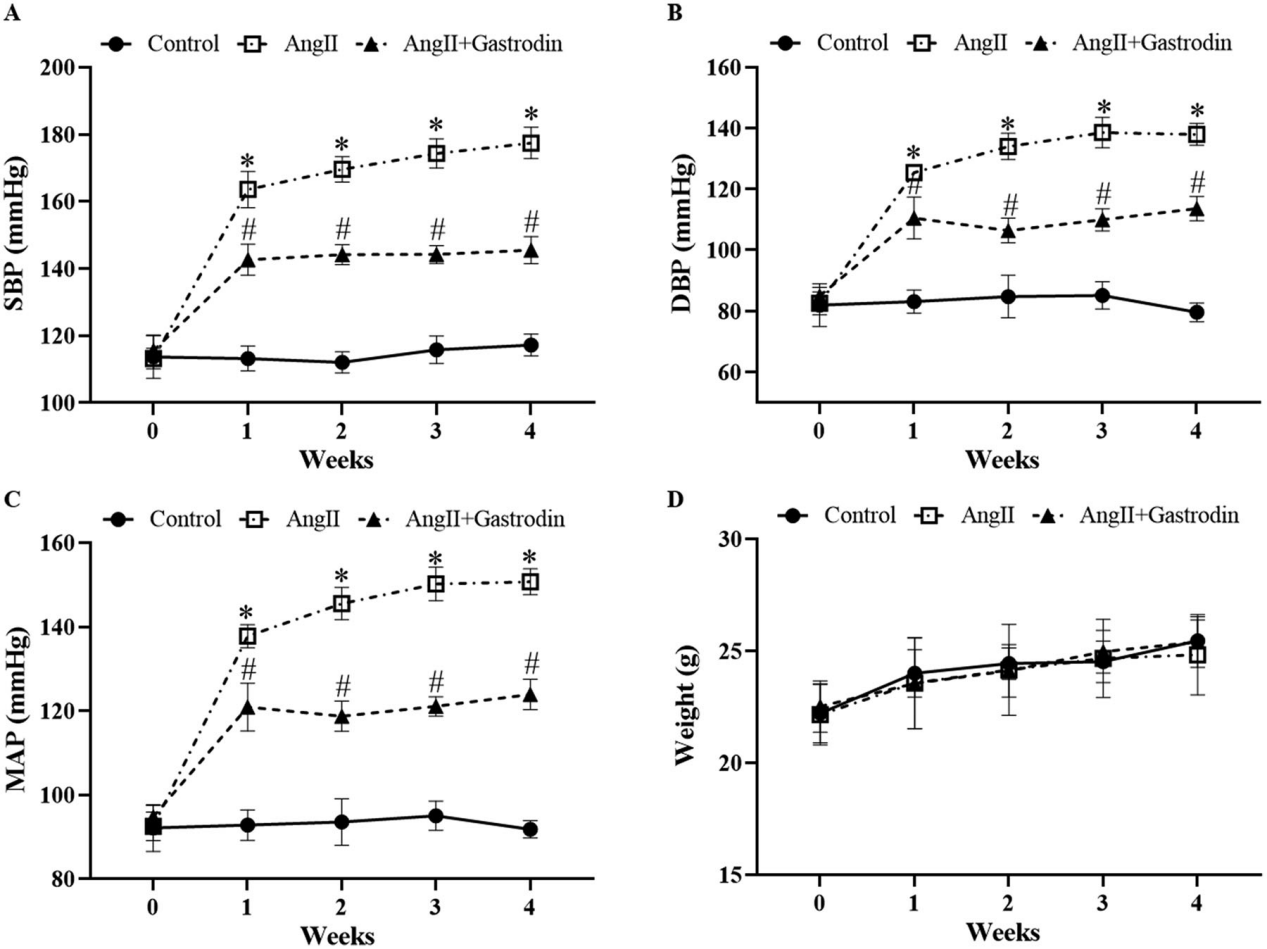
The effects of gastrodin on blood pressure and body weight in angiotensin II (Ang II)-infused mice. A tail-cuff plethysmograph method was used to measure blood pressure in mice once a week for 4 weeks, including (A) systolic blood pressure (SBP), (B) diastolic blood pressure (DBP) and (C) mean arterial pressure (MAP). (D) Body weights of mice in each group. All values are presented as mean ± standard deviation (SD). *p < 0.05 Ang II vs. control group; ^#^*p* < 0.05 Ang II + gastrodin vs. Ang II group.

Systolic blood pressure (SBP), diastolic blood pressure (DBP) and mean arterial pressure (MAP) were markedly elevated in Ang II-infused mice, and all were attenuated by gastrodin treatment (Figure 1(A–C)). No significant differences in body weight were observed between groups (Figure 1(D)).

### Gastrodin alleviates vascular pathological changes in hypertensive mice

**Figure 2.**
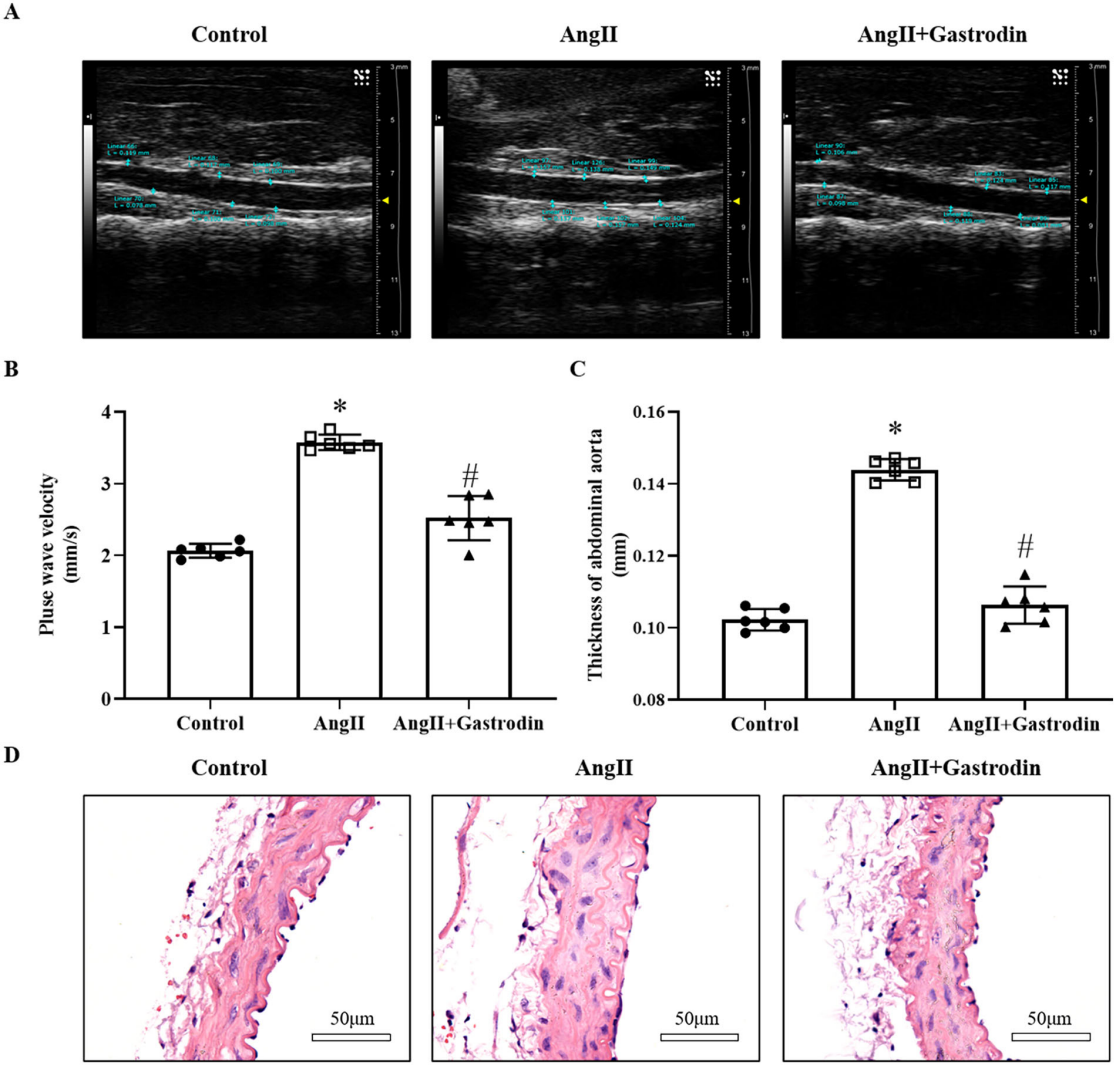
The effects of gastrodin on vascular function and pathological changes in the abdominal aorta of Ang II-infused mice. Ultrasound and haematoxylin and eosin (H&E) staining were performed to determine pulse wave velocity (PWV) and thickness of the abdominal aorta wall in each group. (A) PWV of the abdominal aorta. (B) Thickening of the abdominal aortic wall measured by ultrasound. (C) Representative ultrasonography images of the abdominal aorta. (D) Representative cross-sections of abdominal aortic tissues stained with H&E. Images were taken at a magnification of ×400. All values are presented as mean ± SD. *p < 0.05 Ang II vs. control group; ^#^*p* < 0.05 Ang II + gastrodin vs. Ang II group.

The PWV of the abdominal aorta in Ang II-infused hypertensive mice was increased in the fourth week compared to the control group (3.57 ± 0.16 mm/s vs. 2.06 ± 0.09 mm/s). The PWV of the abdominal aorta was reduced following gastrodin treatment in the fourth week (2.51 ± 0.31 mm/s vs. 3.57 ± 0.16 mm/s; Figure 2(A,B)). Moreover, the thickness of the abdominal aorta was greater in Ang II-infused hypertensive mice compared to controls, and this difference was reversed after gastrodin treatment (Figure 2(C)). Changes in the thickness of the abdominal aorta were confirmed by HE staining (Figure 2(D)).

### Identification of DETs and enrichment of pathways involved in hypertension following gastrodin treatment

**Figure 3.**
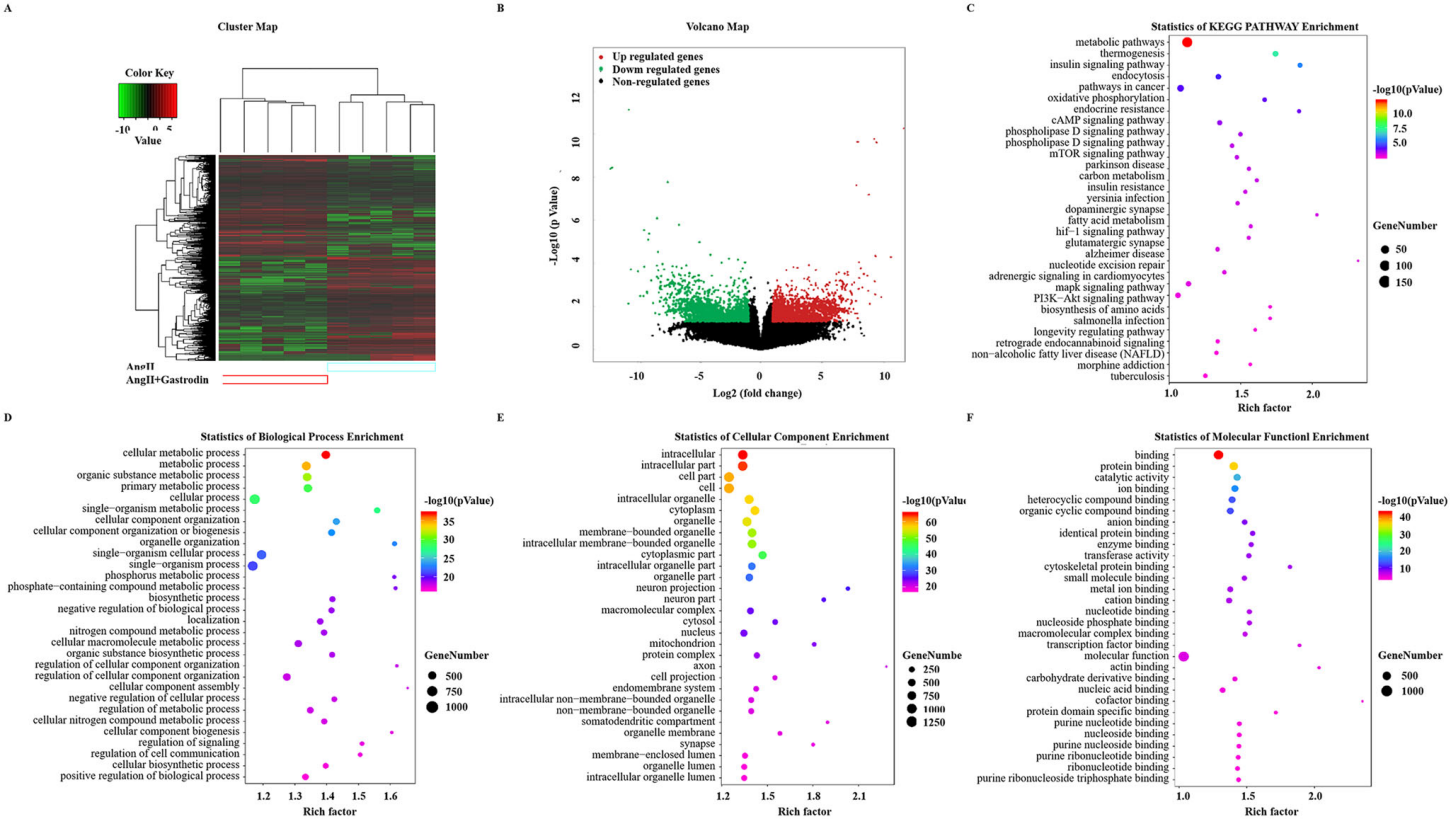
Gene Ontology (GO) enrichment and Kyoto Encyclopedia of Genes and Genomes (KEGG) signalling pathways analysis. (A) Hierarchical clustering plots and (B) volcano plots were used to compare gene expression profiles between the Ang II + gastrodin and Ang II groups (|fold change| ≥ 2, *p < 0.05). (C) KEGG pathway enrichment analysis of differentially expressed transcripts (DETs) comparing the Ang II + gastrodin and Ang II groups. (D–F) GO analysis was performed based on DETs from comparisons of the Ang II + gastrodin and Ang II groups. The top 30 enriched items for (D) biological processes, (E) cellular composition and (F) molecular function are presented.

Treatment with gastrodin resulted in 1377 up-regulated transcripts and 1408 down-regulated transcripts in the abdominal aorta of Ang II-infused hypertensive mice (NCBI GEO: GSE174386). DETs are shown in a cluster map (Figure 3(A)) and volcano map (Figure 3(B)).

KEGG analysis of DETs between the Ang II + gastrodin and Ang II groups identified multiple enriched pathways, including vascular smooth muscle contraction and calcium signalling pathways (Figure 3(C)). The top 30 enriched Gene Ontology (GO) terms are shown in Figure 3(D–F). GO terms were predominantly involved in cellular metabolic processes and regulation of signalling (Figure 3(D)). Cell component analysis revealed that DETs were concentrated in the intracellular, cell part and intracellular organelles (Figure 3(E)). The predominant molecular functions of the top 30 enriched items were protein binding, ion binding and catalytic activity (Figure 3(F)).

### Gastrodin attenuates vasoconstriction and promotes vasodilation of abdominal aortic rings

**Figure 4.**
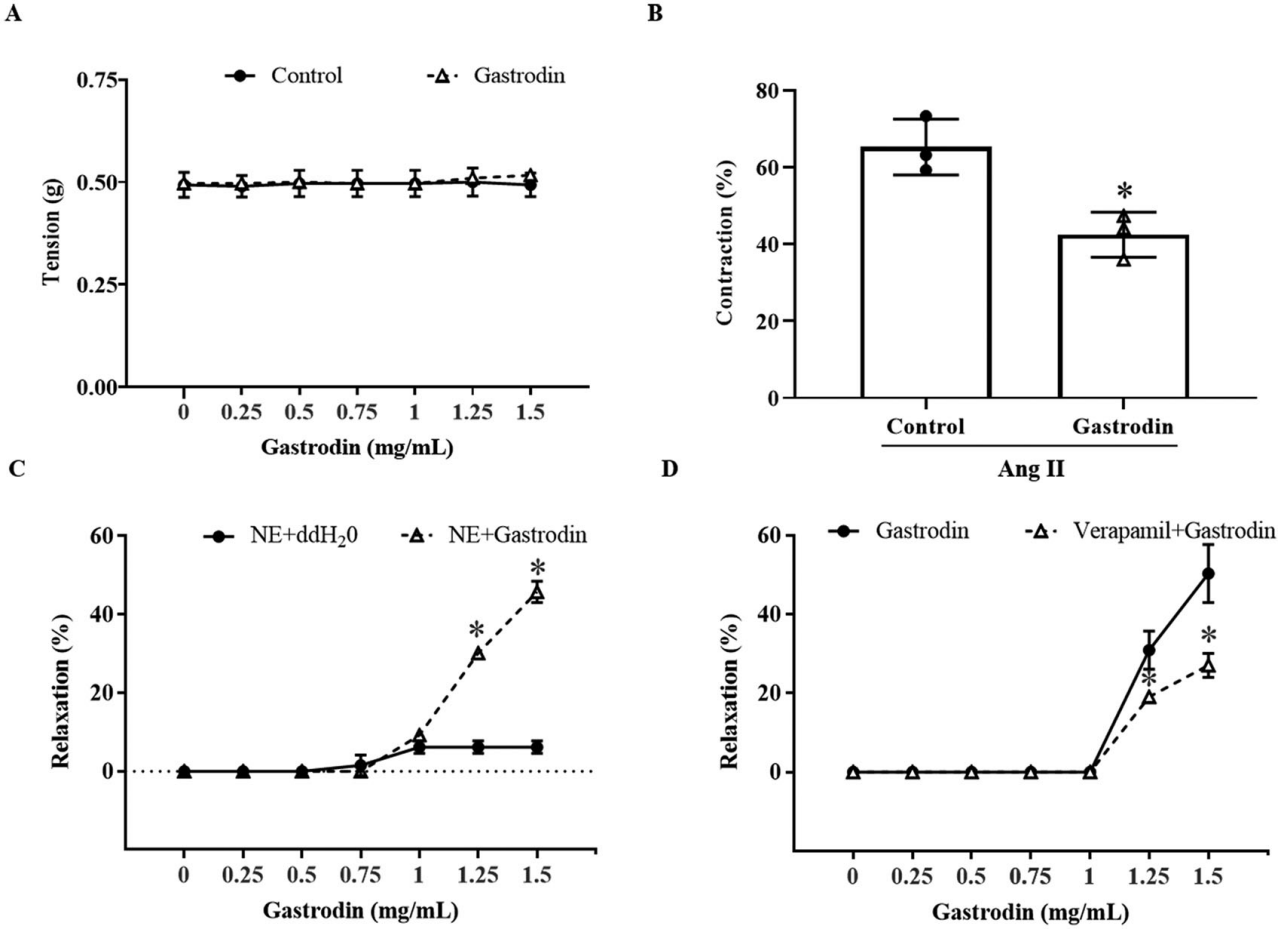
The effects of gastrodin on contraction and relaxation of the abdominal aorta. An Automated Multi Myograph System was performed to measure vasorelaxation of abdominal aortic rings. (A) Vascular contraction at baseline after gastrodin treatment. (B) The effect of gastrodin on Ang II-induced contraction in abdominal aortic rings. (C) The effect of gastrodin on relaxation of abdominal aortic rings precontracted with norepinephrine (NE). (D) The effect of verapamil on gastrodin-induced vasorelaxation. All values are represented as mean ± SD; *p < 0.05 vs. control group.

No change in the tension of abdominal aortic rings was observed after the direct application of cumulative concentrations of gastrodin (Figure 4(A)). Pre-treatment with gastrodin at 1.25 mg/mL dramatically reduced constriction of abdominal aortic rings in response to Ang II (vasoconstriction percentage: 42.50 ± 5.85% vs. 65.25 ± 7.26%, *p < 0.05; Figure 4(B)). Furthermore, treatment with 1.25 or 1.5 mg/mL gastrodin resulted in relaxation of abdominal aortic rings precontracted with NE (vasodilation percentage in response to 1.25 and 1.5 mg/mL of gastrodin: 30.13 ± 1.35% and 45.76 ± 2.73%, respectively, *p < 0.05; Figure 4(C)). The vasodilative effect of gastrodin was impaired by pre-treatment with verapamil, a L-type calcium channel inhibitor (vasodilation percentage of gastrodin vs. verapamil + gastrodin: 30.90 ± 4.81% vs. 19.07 ± 0.90% (1.25 mg/mL) and 50.30 ± 7.35% vs. 27.03 ± 3.10% (1.5 mg/mL), respectively, *p < 0.05; Figure 4(D)).

### Gastrodin suppresses vascular contraction pathways activated by Ang II

**Figure 5.**
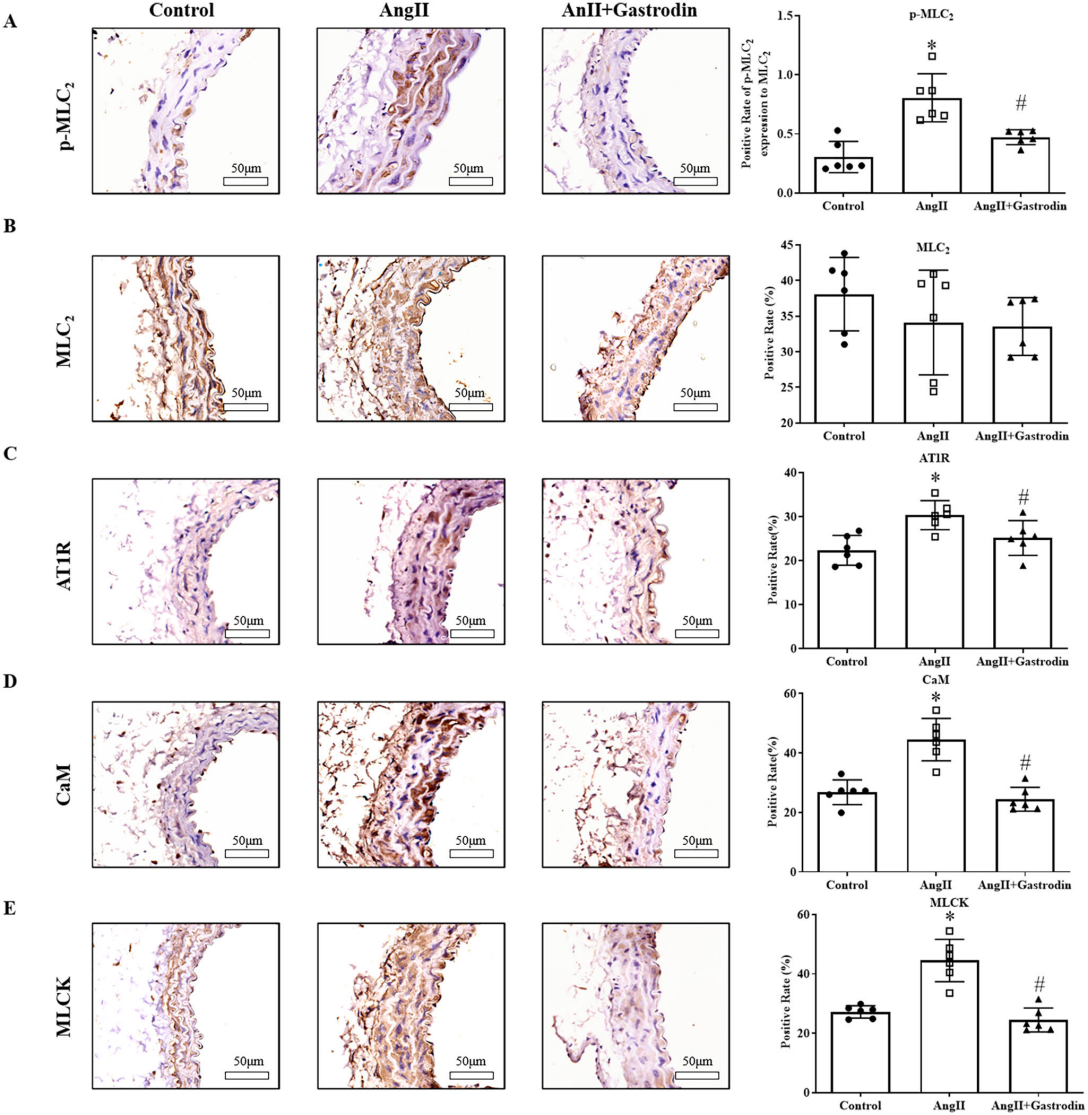
Expression of proteins involved in the MLCK/p-MLC_2_ pathway in abdominal aorta tissues. Immunohistochemical (IHC) staining was used to detect protein levels of (A) phospho-myosin light chain 2 (p-MLC_2_), (B) myosin light chain 2 (MLC_2_), (C) angiotensin type 1 receptor (AT1R), (D) calmodulin (CaM) and (E) myosin light chain kinase (MLCK) in abdominal aorta tissues. Images were taken at a magnification of ×400. All values are represented as mean ± SD; *p < 0.05 Ang II vs. control group; ^#^*p* < 0.05 Ang II + gastrodin vs. Ang II group.

Significant up-regulation of p-MLC_2_ was observed in the abdominal aorta of Ang II-infused mice, with decreased expression of p-MLC_2_ observed after gastrodin treatment (Figure 5(A,B)). Moreover, the expression of the upstream molecules, including AT1R, CaM and MLCK, was similar to that of p-MLC_2_ (Figure 5(C–E)).

### Gastrodin inhibits intracellular calcium release induced by Ang II in VSMCs

**Figure 6.**
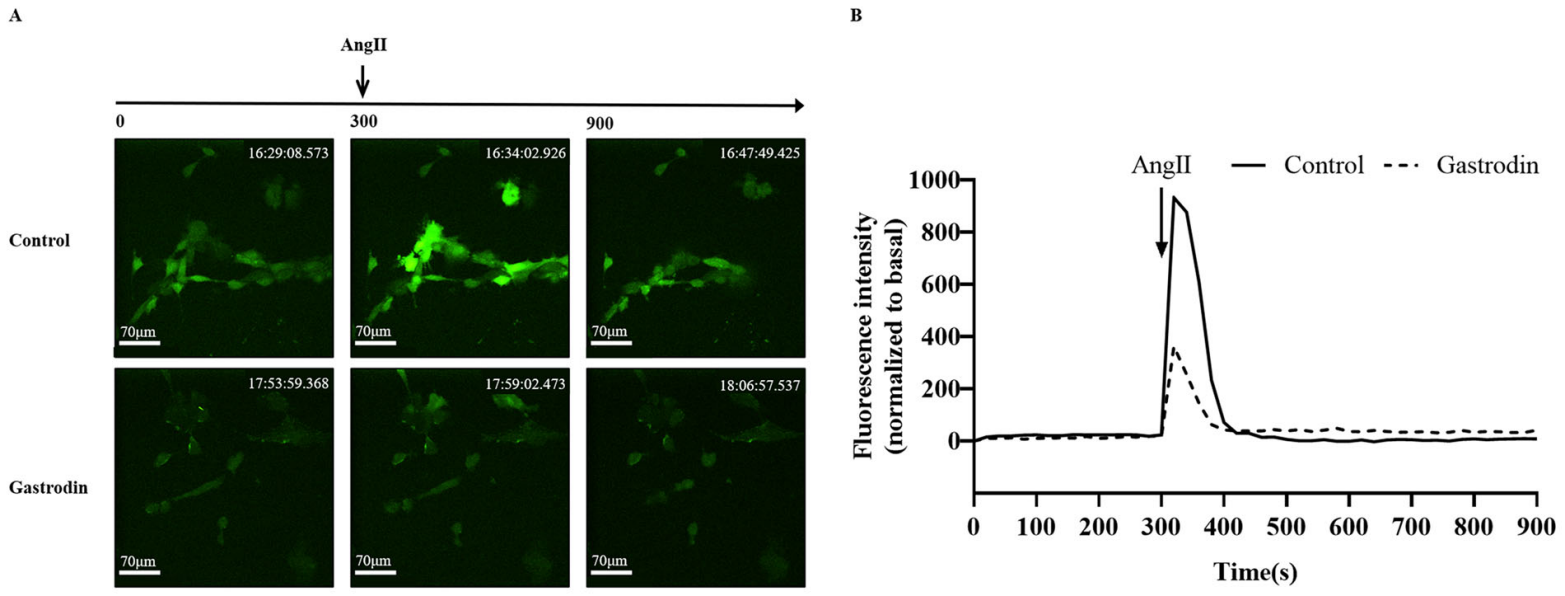
The effects of gastrodin on release of intracellular calcium in Ang II-stimulated vascular smooth muscle cells (VSMCs). Confocal microscopy was performed to measure changes in intracellular Ca^2+^ concentration in real time. (A) Representative images of intracellular calcium release in VSMCs at different time points. Images were taken at a magnification of ×200. (B) A graph of the change in intracellular Ca^2+^ concentration is shown.

Primary VSMCs were isolated and characterized according to α-SMA expression (Supplementary Figure S1A). CCK-8 analysis demonstrated that the viability of VSMCs was significantly reduced following treatment with 0.01–10 μM Ang II (Supplementary Figure S1B) and unaffected by treatment with 0–200 μM of gastrodin (Supplementary Figure S1C). Accordingly, 1 μM of Ang II and 50, 100 or 200 μM of gastrodin were selected for subsequent studies. The release of intracellular Ca^2+^ in VSMCs indicated that gastrodin treatment partially reversed the release of intracellular Ca^2+^ stimulated by Ang II (Figure 6).

### Gastrodin attenuates activation of MLCK/p-MLC_2_ pathway in VSMCs

**Figure 7.**
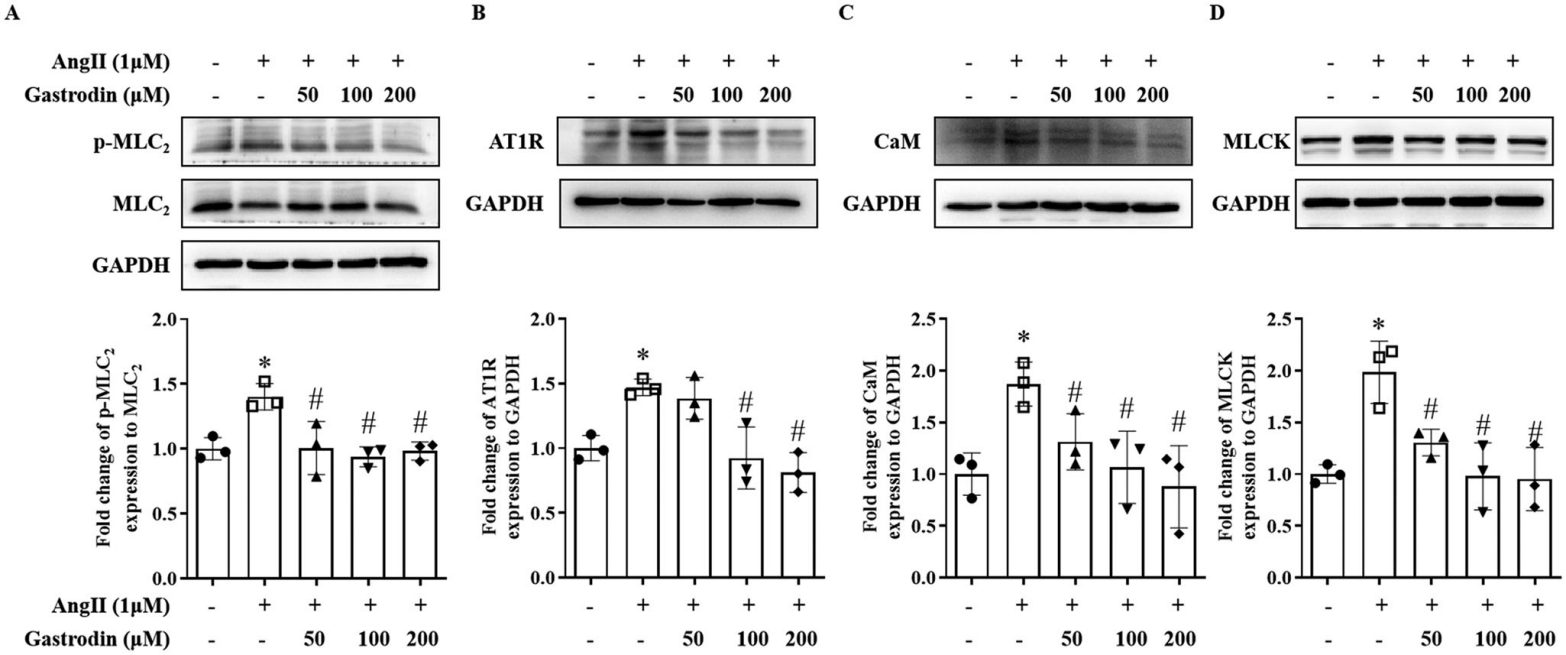
The effects of gastrodin on the MLCK/p-MLC_2_ pathway in Ang II-stimulated VSMCs. Western blotting was used to determine protein levels of (A) p-MLC_2_, (B) AT1R, (C) CaM and (D) MLCK in Ang II-stimulated VSMCs pretreated with gastrodin (50, 100 or 200 μM) for 24 h. All values are represented as mean ± SD; *p < 0.05 Ang II vs. control group; ^#^*p* < 0.05 vs. Ang II group.

Western blotting demonstrated increased protein levels of AT1R, MLCK, CaM and p-MLC_2_ following stimulation of Ang II in VSMCs, with pre-treatment with gastrodin significantly attenuating the effect of Ang II (Figure 7).

## Discussion

Gastrodin is a natural compound extracted from *Gastrodia elata*. Extensive studies of the pharmacological properties of gastrodin have demonstrated a therapeutic effect on reducing blood pressure in patients from Chinese populations (Zhang et al. 2008; Qian et al. 2020). Moreover, gastrodin has been shown to inhibit the RAAS and PPARγ and decrease SBP in SHRs (Liu et al. 2015). However, the effect of gastrodin treatment has not previously been reported in Ang II-infused hypertensive mice. The present study provides novel evidence that gastrodin treatment lowers blood pressure in hypertensive mice, but it still has some limitations for only one dose of gastrodin used in our study.

Abnormalities in vascular function and structure underlie the initiation and development of hypertension (Schiffrin 2012). Previous studies have revealed that gastrodin treatment has a protective effect on blood vessels in vascular dementia (Li et al. 2022) and diabetes-induced cerebellar alterations (Zhang et al. 2020). However, few studies have evaluated the effect of gastrodin on vascular structural changes and dysfunction caused by hypertension. The ultrasound and H&E staining results in the present study demonstrate that gastrodin treatment reduces the thickness and PWV of the abdominal aorta, indicating gastrodin reduces vascular pathological changes and dysfunction in hypertensive mice. In addition, augmented vasoconstriction is a pathological characteristic of hypertension, typically due to increased vasoconstriction mediated by vascular smooth muscle (Touyz et al. 2018). The identification of DETs and KEGG analysis following gastrodin treatment demonstrated enrichment of vascular smooth muscle contraction signalling pathways, highlighting the role of gastrodin treatment on vasoconstriction and vasorelaxation. Our *ex vivo* studies of vascular tension revealed that gastrodin causes vasorelaxation of abdominal aortic rings precontracted with NE and ameliorates Ang II-induced vasoconstriction, which corroborate previous studies reporting the vasorelaxant effect of gastrodin on isolated thoracic aorta rings (Xie et al. 2015) and mesenteric artery rings (Chen et al. 2017) in rats.

In the pathways enriched following gastrodin treatment, intracellular Ca^2+^ is an important secondary messenger that triggers the contraction of smooth muscle cells (House et al. 2008; Anjum 2018; Fan et al. 2019). Increased intracellular Ca^2+^ activates the MLCK/p-MLC_2_ pathway, which has been identified as a key signalling pathway that promotes vasoconstriction leading to hypertension (Jernigan et al. 2008; Iizuka et al. 2011; Wang et al. 2015; Liu et al. 2021; Wei et al. 2021). In the present study, we also observed that gastrodin treatment suppressed the release of intracellular Ca^2+^ in VSMCs and the activation of the MLCK/p-MLC_2_ signalling pathway *in vivo* and *in vitro*. These findings indicate that improving dysfunctional vascular smooth muscle contraction by suppressing the MLCK/p-MLC_2_ pathway may represent one of the mechanisms underlying the therapeutic effects of gastrodin on hypertension. However, future studies are required to fully determine the regulatory effects of gastrodin treatment on other signalling pathways that are enriched on KEGG analysis following gastrodin treatment.

## Conclusions

The results of the present study demonstrate that gastrodin treatment lowers blood pressure, suppresses Ang II-induced vascular contraction, and inhibits activation of the MLCK/p-MLC_2_ pathway. These findings provide insights into the protective effects of gastrodin on vascular function and its efficacy as a treatment for hypertension.

## Supplementary Material

Supplemental Material

## Data Availability

The datasets used and/or analysed during the current study are available from the corresponding author on reasonable request.
