# Supplementary material for: Gastrodin attenuates angiotensin II-induced vascular contraction and MLCK/p-MLC2 pathway activation
Source: Pharm Biol. 2023 May 21;61(1):858–67. doi: 10.1080/13880209.2023.2207591 (PMC10202000; doi:10.1080/13880209.2023.2207591)
Supplement: Supplemental Material [file IPHB_A_2207591_SM5282.docx]

# Supplementary Figures

## *Figure S1. Identification of the VSMCs and the cell viability of Gastrodin or Ang II treatment.*


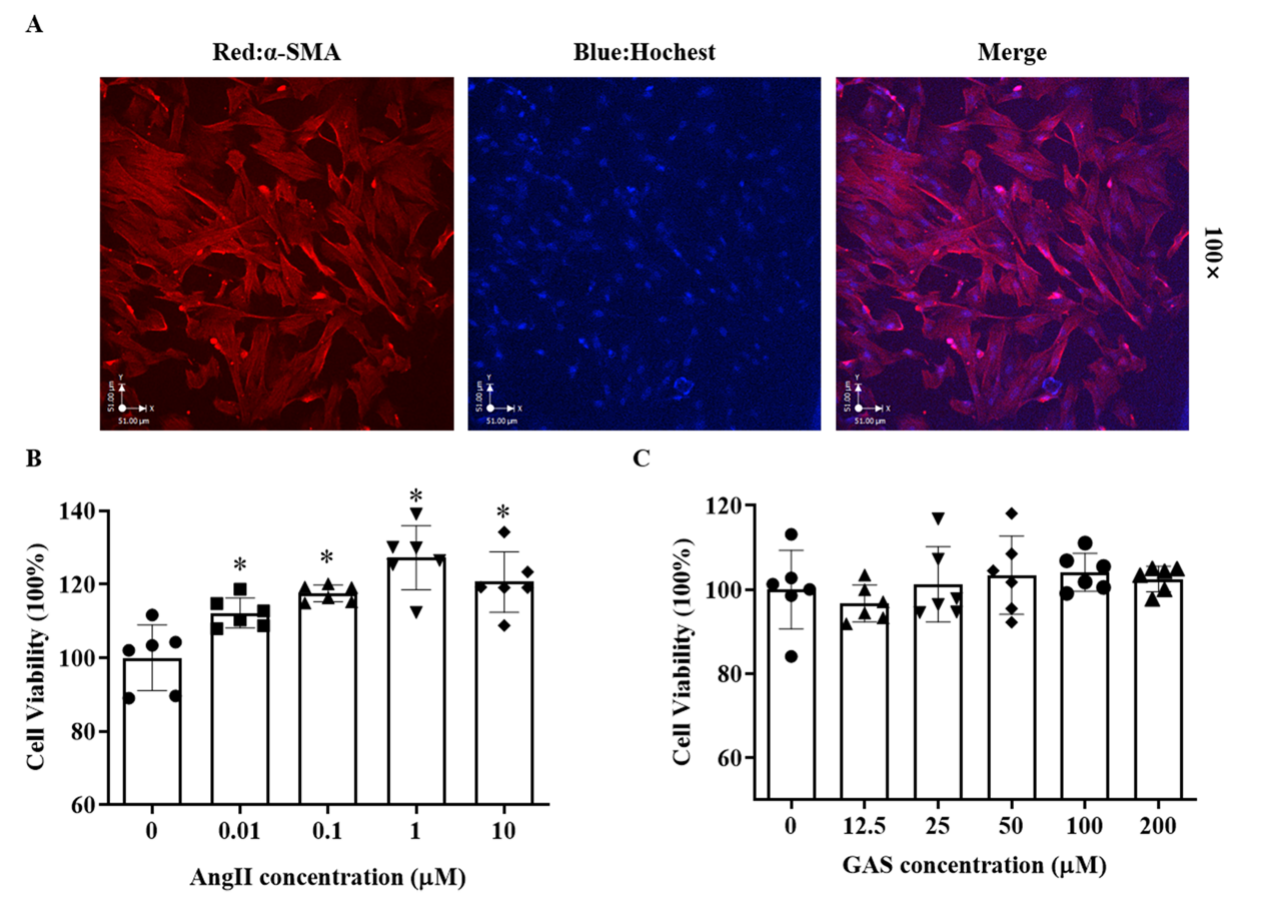


(A) The primary VSMCs were isolated and identified by immunofluorescence staining against α-SMA antibody using confocal microscopy at a magnification of 100×. (B, C) The cell viability of VSMCs after (B) Ang II (0, 0.01, 0.1, 1, 10 μM) or (C) gastrodin (0, 12.5, 25, 50, 100, 200 μM) treatment for 24 h was determined by CCK-8 analysis. Data were normalized to the viability of untreated control cells and set as 100%.
